# Structure and Selected Properties of SnO_2_ Thin Films

**DOI:** 10.3390/ma17133348

**Published:** 2024-07-06

**Authors:** Aneta Kania, Magdalena M. Szindler, Marek Szindler, Zbigniew Brytan, Wojciech Łoński

**Affiliations:** 1 Department of Engineering Materials and Biomaterials, Faculty of Mechanical Engineering, Silesian University of Technology, Konarskiego 18a, 44-100 Gliwice, Poland; magdalena.szindler@polsl.pl (M.M.S.); zbigniew.brytan@polsl.pl (Z.B.); wojciech.lonski@polsl.pl (W.Ł.); 2 Scientific and Didactic Laboratory of Nanotechnology and Material Technologies, Faculty of Mechanical Engineering, Silesian University of Technology, Towarowa 7, 44-100 Gliwice, Poland; marek.szindler@polsl.pl

**Keywords:** SnO_2_ thin films, ALD method, structure analysis, corrosion studies, corrosion resistance

## Abstract

Magnesium and its alloys are attractive temporary implants due to their biocompatibility and biodegradability. Moreover, Mg has good mechanical and osteoinductive properties. But magnesium and Mg alloys have one significant disadvantage: poor corrosion resistance in a physiological environment. Hence, a deposition of various layers on the surface of Mg alloys seems to be a good idea. The purpose of the article is to analyze the structure and morphology of two MgCa2Zn1 and MgCa2Zn1Gd3 alloys coated by SnO_2_ ALD (atomic layer deposition) films of various thickness. The studies were performed using scanning electron microscopy (SEM), X-ray fluorescence (XRF), and an X-ray diffractometer. The corrosion activity of the thin films and substrate alloys in a chloride-rich Ringer’s solution at 37 °C was also observed. The corrosion tests that include electrochemical, immersion measurements, and electrochemical impedance spectroscopy (EIS) were evaluated. The results indicated that SnO_2_ had a heterogeneous crystal structure. The surfaces of the thin films were rough with visible pores. The corrosion resistance of SnO_2_ measured in all corrosion tests was higher for the thicker films. The observations of corrosion products after immersion tests indicated that they were lamellar-shaped and mainly contained Mg, O, Ca, and Cl in a lower concentration.

## 1. Introduction

Metallic biomaterials, such as titanium alloys and stainless steels, play an important role in orthopedic surgery. They have many attractive properties but also have one disadvantage: the necessity of removing implants after they fulfill their role in the body. Magnesium and its alloys are promising materials as temporary implants due to their good biocompatibility and biodegradability [1,2,3]. Magnesium is also an environmentally friendly material with a lower CO_2_ emission compared to other metals, such as aluminum [4]. The mechanical properties of magnesium alloys, for example plasticity and stiffness, are better than the properties of other bioresorbable materials [5,6]. In addition, the density of Mg alloys is approximately 1.74 g cm^–3^, which is close to the density of cortical bone (1.75 g·cm^–3^) [3]. Magnesium also possesses osteoinductive properties, which indicates that it can facilitate fracture healing. Furthermore, it is an optimal material for bone repair. However, the poor corrosion resistance of Mg limits the widespread use of magnesium alloys in medicine. Therefore, it is necessary to slow down the rate of degradation of the alloys to fit the rate of newly formed bone near the implants [7,8].

In addition, it is crucial that such metals form thin protective passive oxide films that act as a barrier separating the metal from the physiological environment. The alloys utilized in biomedical applications, such as titanium and stainless steel, are capable of forming passive surface oxide films within the human body, which typically provide satisfactory corrosion resistance. However, if the oxide film is damaged, this can lead to complications. 

The poor corrosion resistance of Mg alloys is due to the fact that magnesium is an active metal and thus susceptible to corrosion. The naturally occurring passive film of MgO on the surface of Mg alloys is very thin (approx. 1 nm) and provides only poor protection against corrosion [8]. The Mg alloys in a physiological environment degrade as a result of electrochemical reactions, resulting in the formation of magnesium hydroxide, hydrogen, and other compounds [7,8,9]. Therefore, surface modification is an effective and economical strategy to improve surface properties by building functional films/coatings on their surface [10,11,12]. In recent years, a large number of studies have been presented related to corrosion resistance of various coatings/layers, e.g., TiO_2_ [13,14] and ZnO [15,16] deposited on the surface of Mg and its alloys. Among these materials, there are a few studies on the use of SnO_2_ [17,18] coatings. These surface coatings/layers can be applied using different deposition methods, such as sol-gel, PVD (physical vapor deposition), which includes evaporation, sputtering, and CVD (chemical vapor deposition). There are many variants of CVD, with one of the methods being the ALD (atomic layer deposition) technique. It is unique because the precursor and reagent are introduced separately into the chamber, reacting only on the surface of the coated element. This method offers a number of advantages. It does not require high temperature or high vacuum. ALD is capable of regulating the thickness of materials at the molecular level, enabling the uniform coating of elements with complex topographies. The produced layers exhibit homogeneity with respect to their optical and electrical properties. Additionally, the coating produced by ALD adheres to the surface shape, which is particularly advantageous in applications such as bone surgery where precise adherence is crucial. Furthermore, by controlling the temperature and the number of application cycles, it is possible to obtain layers of varying thicknesses and properties.

Tin has been used in medicine since the 19th century. It is known that Sn is an important and vital element in the body. It is non-toxic and has a high biosafety profile [3]. It should be mentioned that Sn is easily passivated, most often spontaneously, and it also has an absence of oxidants. However, in the literature, there have been limited studies on the modification of magnesium alloys by using Sn.

Tin dioxide (SnO_2_) exhibits a dominant tetragonal crystal structure, which is analogous to that of titanium dioxide (TiO_2_). It is therefore possible that SnO_2_ may have a similar effect to TiO_2_ in inducing the formation of a calcium phosphate layer [19]. Furthermore, it was found that tin dioxide doped with nanocrystals has antibacterial activity [20,21]. In the studies presented by Cui et al. in [20], SnO_2_-doped Ca-P coating was applied on AZ31 alloy using hydrothermal deposition. The results demonstrated that the coating exhibited a globular morphology with a long lamellar crystalline structure. By increasing the addition of SnO_2_ nanoparticles, the coating surface became smoother. The corrosion current density (j_corr_) and the volume of hydrogen evolution of the coating with SnO_2_ in Hank’s solution were decreased in comparison with the coating without nano-sized particles of SnO_2_ and the uncoated alloy. These indicate an improvement in the corrosion resistance of the SnO_2_-doped coating. Wang et al. [17] studied the corrosion behavior of CVD-deposited fluorine-doped SnO_2_ films on the surface of 317 L stainless steel. The researchers observed a small and extremely stable current from the SnO_2_ in the 1 M H_2_SO_4_ + 2 ppm F^−^ solution at a temperature of 70 °C. They stated that the coating improved the corrosion resistance of the base alloy. An improvement of the corrosion rate of Mg-Y-RE (WE) alloy deposited with an SnO_2_ coating by magnetron sputtering was also presented by Jin et al. [11]. The authors in [22] added SnO_2_ nanoparticles to SiO_2_ coating and deposited them on A36 carbon steel using sol-gel and dip-coating methods. The investigation showed that all applied coatings (with SnO_2_ content of 0.1, 2.5, 5.0, and 7.5% vol.) improved the corrosion resistance of the A36 steel in a 3 wt.% NaCl solution at room temperature. It was also found that tin dioxide nanoparticles influenced the sol-gel SiO_2_ film formation and the coated samples’ stability in a corrosive environment.

Due to the limited number of studies on the corrosion resistance of tin oxide films applied to magnesium alloys, the objective of this work was to evaluate the effectiveness of SnO_2_ thin films with different thicknesses (35, 52.5, and 70 nm) deposited on the surfaces of MgCa2Zn1 and MgCa2Zn1Gd3 alloys in improving the corrosion resistance of the substrate alloys. In the context of this research, the authors will analyze the morphology of SnO_2_ films obtained using the atomic layer deposition (ALD) method before and after the corrosion tests. The corrosion activity of the thin films will be tested in an aggressive Ringer’s solution, which simulates body fluid. 

## 2. Materials and Methods

The MgCa2Zn1 and MgCa2Zn1Gd3 alloys were substrate materials for the deposition of thin SnO_2_ films. The Mg-based alloys were prepared using high-purity metals: magnesium (99.99%), calcium (99.5%), zinc (99.99%), and gadolinium (99.9%). The alloys were cast in a medium-frequency induction furnace (PI25 model, Elkon, Rybnik, Poland) at a temperature of 750 °C, with argon as the protective gas. In order to facilitate the ALD process, samples were prepared in the form of cylinders with a diameter of 13 mm and a height of 6 mm. All samples were mechanically polished with SiC paper on a grinder and polisher (LaboPol-25 model, Struers, Ballerup, Denmark) with a rotational speed of 150 rpm. The following grade order was used: firstly 500, then 800, then 1200, and finally 4000, and then polished with a diamond suspension using MD-Nap. Subsequently, the samples were subjected to an ultrasonic degreasing process in acetone for a period of 10 min. Following this, they were cleaned in alcohol and washed with distilled water.

The SnO_2_ thin film was deposited via atomic layer deposition (ALD) using a Picosun R 200 reactor (Espoo, Finland). The tin chloride was used as the precursor, while deionized water served as the reagent. For the selected compounds, the thermal ALD parameters were used, with a deposition temperature of 300 °C and pulse lengths of 0.1 and 4 s, respectively, to generate the precursor and water. A purging step with nitrogen gas flowing for four seconds was used between pulses to remove any remaining precursors and reaction by-products. The number of cycles for the tin dioxide thin films ranged from 1000 to 2000.

The thickness of the prepared thin films was determined using an FR-pRo-UV/VIS optical reflectometer (ThetaMetrisis SA., Peristeri, Greece). The reflected light technique was employed for the measurements. Reflectometric measurements are based on the theory of total reflection. This value represents the ratio of reflected light to incident light. A light beam falls on the sample surface, where it is reflected from the top and bottom of the thin film. Subsequently, the beam is directed to the CCD array via an optical fiber and processed on a computer. The result is a spectrogram of linear interference oscillations proportional to the thickness of the thin film, which is displayed on the monitor.

The structure and morphology of the samples were characterized using a scanning electron microscope (SEM) (Zeiss, SUPRA 35 model; EHT = 5.0 and 10 kV, SE mode, in-lens detector) equipped with an energy-dispersive X-ray spectroscopy (EDS) detector. The SnO_2_-coated Mg-based alloys and the corrosion products resulting from the corrosion tests were identified using EDS analysis of the samples’ surfaces.

Additional tests on the chemical composition of the deposited thin films were carried out using the SHIMADZU (Kioto, Japan) EDX-7000 X-ray fluorescence spectrometer. X-ray fluorescence spectrometry (XRF) is an analytical technique that uses the characteristic X-ray radiation emitted by elements as a result of excitation with higher energy X-rays. At 4 kV (4000 eV), the wavelength is approximately 0.31 nm. At 50 kV (50,000 eV), the wavelength is approximately 0.025 nm. Thus, the EDX-7000 can detect X-ray wavelengths in the range of about 0.025 to 0.31 nm (25 to 310 pm).

Phase analysis of the SnO_2_ thin films was conducted using a PANalytical X’Pert PRO X-ray diffractometer (PANalytical, Almelo, The Netherlands) with Co Kα radiation. The analysis was performed with step registration over a 2θ angular range of 20 to 110°. Qualitative X-ray analysis was conducted using HighScore Plus software v. 3.0e, which employs a dedicated PAN-ICSD phase identification card database.

Corrosion studies were conducted using electrochemical and immersion tests. Electrochemical studies were performed on an Autolab PGSTAT302N Multi BA potentiostat (Metrohm AG, Herisau, Switzerland). Measurements were made in a chloride-rich Ringer’s solution (8.6 g·dm^−3^ NaCl, 0.3 g·dm^−3^ KCl, 0.48 g·dm^−3^ CaCl_2_·6H_2_O) at a temperature of 37 °C. The corrosion potential scan rate was set at 1 mV·s^–1^. Polarization curves with Tafel extrapolation were determined after a stabilization period of 5 min. The corrosion parameters (e.g., corrosion potential—E_corr_; corrosion current density—j_corr_; and corrosion polarization resistance—R_p_) were then determined. 

In order to determine the electrical characteristics of the SnO_2_ films, electrochemical impedance spectroscopy (EIS) measurements were also conducted at a temperature of 24 °C. The investigation was conducted by recording changes in resistance and impedance in the variable frequency range from 100 kHz to 0.01 Hz using a 10 mV signal in Ringer’s solution. The results were used to determine the Bode and Nyquist relationships.

The immersion tests of the samples were performed in Ringer’s solution at 37 °C for 48 h. The measurements provided an estimation of the gas corrosion product (H_2_ evolution volume). Cylindrical samples with a testing area of 1.3 cm^2^ were prepared for the measurements. The volume of evolved H_2_ was quantified in relation to the frontal area of the samples. 

Following immersion testing, the corroded surfaces of the MgCa2Zn1 and MgCa2Zn1Gd3 alloys with SnO_2_ films were observed using scanning electron microscopy.

## 3. Results and Discussion

**Figure 1:**
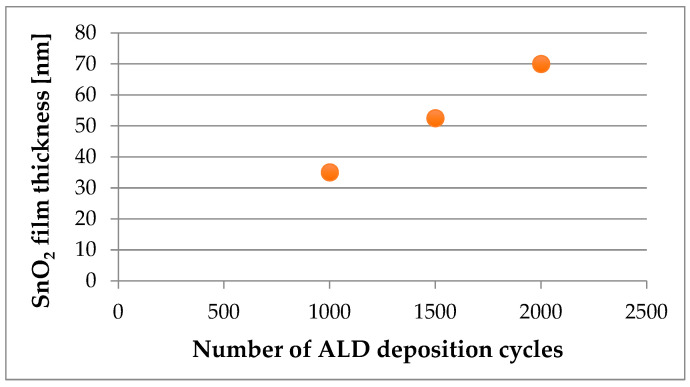
Relationship between SnO_2_ film thickness and the number of ALD deposition cycles.

The SnO_2_ thin films were applied to MgCa2Zn1 and MgCa2Zn1Gd3 alloys after 1000, 1500, and 2000 deposition cycles, respectively. The SnO_2_ thin films were prepared with a number of cycles ranging from 1000 to 2000, and their properties were subsequently measured. The thicknesses of the thin films were confirmed using optical reflectometer. The thicknesses measured in the reflection mode were 35, 52.5, and 70 nm, respectively (Figure 1). Based on this, the average growth speed of the SnO_2_ layer was determined to be 0.035 nm per cycle. 

**Figure 2:**
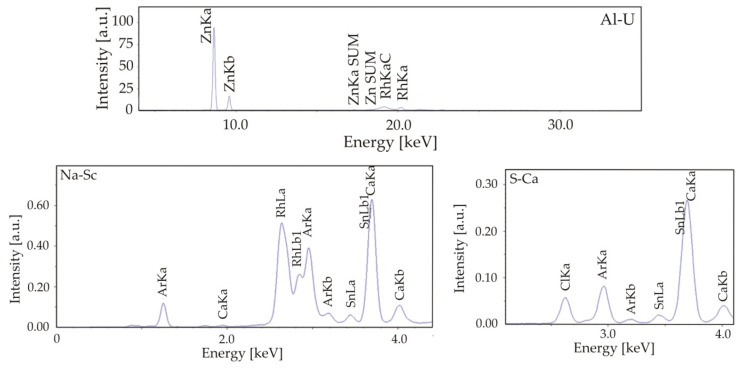
Dispersive X-ray fluorescence spectra of MgCa2Zn1 sample coated with the SnO_2_ layer after 2000 cycles.

X-ray fluorescence spectrometry (XRF) tests confirmed the presence of Sn on the surface of MgCa2Zn1 and MgCa2Zn1Gd3 samples. An example spectrum for a MgCa2Zn1 sample coated with a SnO_2_ layer after 2000 cycles is shown in Figure 2. 

**Figure 3:**
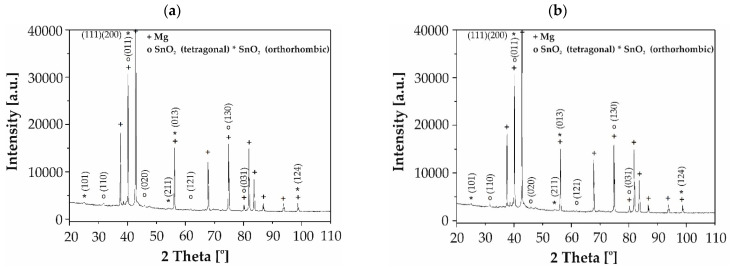
X-ray diffraction patterns of the SnO_2_ films (70 nm thick) deposited on (**a**) MgCa2Zn1 and (**b**) MgCa2Zn1Gd3 alloys.

Furthermore, the results of X-ray phase analysis verified that SnO_2_ phases in two varieties of the crystal lattice—tetragonal and orthorhombic—were detected in the studied thin films applied to MgCa2Zn1 and MgCa2Zn1Gd3 alloys (Figure 3). The diffraction patterns showed characteristic peaks at 31.544, 40.035, 45.213, 61.958, 74.860, and 79.945 degrees of the 2θ angle for the SnO_2_ assigned to the tetragonal crystal lattice (JCPDS card No. 98-005-6674) and also showed peaks at 24.794, 39.848, 40.086, 53.732, 56.020, and 98.870 degrees of the 2θ angle for the SnO_2_ assigned to the orthorhombic crystal lattice (JCPDS card No. 98-018-1282) deposited onto Mg-based alloys. The phase analysis indicates that the SnO₂ has a dominated rutile structure (space group P 42/m n m; space group no. 136; lattice parameters: a = 4.6540 Å, b = 4.6540 Å, c = 3.1580 Å).

**Figure 4:**
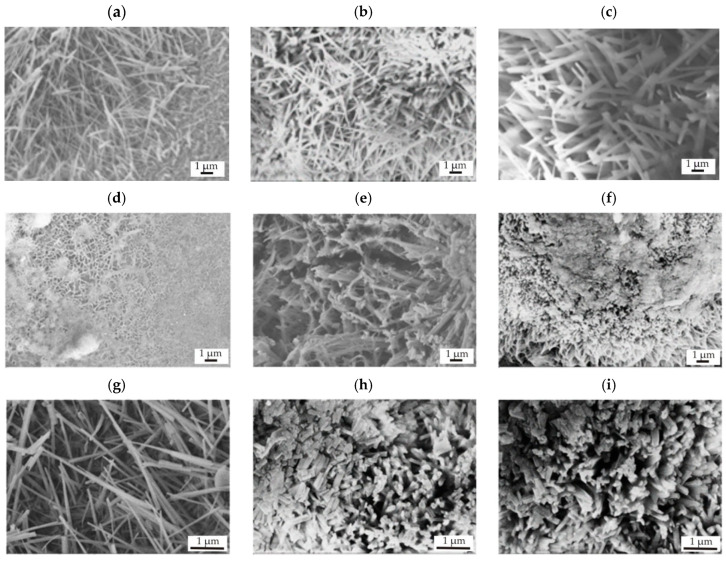
SEM images of SnO_2_ thin films deposited on MgCa2Zn1 alloy after (**a**) 1000, (**b**) 1500, and (**c**) 2000 cycles and on MgCa2Zn1Gd3 alloy after (**d**,**g**) 1000, (**e**,**h**) 1500, and (**f**,**i**) 2000 cycles.

The SEM observations showed that the surface morphologies deposited on Mg-based alloys were very similar. Figure 4 shows images of coatings of different thicknesses applied to the MgCa2Zn1 and MgCa2Zn1Gd3 alloys. It can be observed that the SnO_2_ films exhibited a heterogeneous structure and a lamellar-like shape [20] composed of nanorods forming bundles [23]. Extending the ALD deposition time results in the formation of shorter and thicker SnO_2_ crystallites that agglomerate and form bundles oriented in different directions. All surfaces were rough with visible pores. In addition, the lamellae became thicker as the thickness of the SnO_2_ film increased.

**Figure 5:**
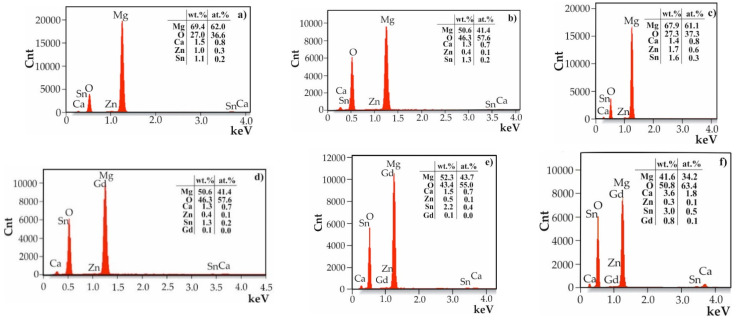
EDS analysis of the SnO_2_ thin films deposited onto MgCa2Zn1 alloy after (**a**) 1000 cycles, (**b**) 1500 cycles, and (**c**) 2000 cycles and onto MgCa2Zn1Gd3 alloy after (**d**) 1000 cycles, (**e**) 1500 cycles, and (**f**) 2000 cycles.

Figure 5 presents the results of the EDS analysis for the SnO₂ thin films with various thicknesses applied to MgCa2Zn1 and MgCa2Zn1Gd3 alloys. The analysis indicated that the surfaces of the samples contained Mg, Ca, Zn, and Gd elements from the substrate alloys and Sn and O from the thin films. It can be observed that the Sn content increases in a uniform manner as the layer thickness increases, whereas the tin content is lower for the alloy that does not contain gadolinium (Figure 5a–c). 

**Figure 6:**
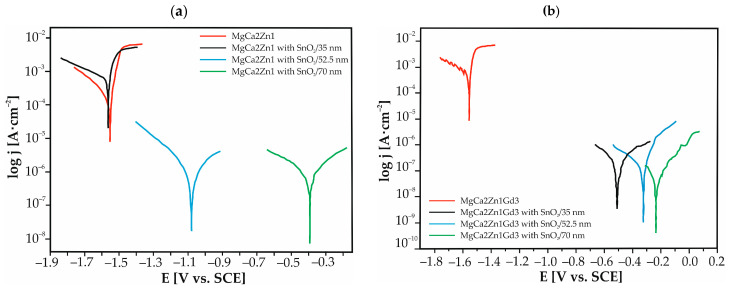
Polarization curves for the SnO_2_ thin films and uncoated Mg-based alloys in Ringer’s solution at 37 °C: (**a**) MgCa2Zn1 alloy; (**b**) MgCa2Zn1Gd3 alloy.

Electrochemical tests were conducted in Ringer’s solution at a temperature of 37 °C. The potentiodynamic curves for SnO_2_ thin films and uncoated Mg alloys are presented in Figure 6 and show similar shapes. The corrosion potential, E_corr_, of the SnO_2_ films after different numbers of deposition cycles exhibited a shift to more positive values in comparison to the uncoated Mg alloys (the exception was the 35 nm-thick layer deposited to MgCa2Zn1, where the potential was slightly lower than those of the uncoated alloy). In general, thicker films had lower potential. This suggests an improvement in the corrosion resistance of MgCa2Zn1 and MgCa2Zn1Gd3 alloys when coated with SnO_2_ films. Moreover, the curves for SnO_2_ films on MgCa2Zn1 were observed to be located in a higher current range (10^−3^–10^−5^A·cm^−2^) than those of the same oxides deposited on MgCa2Zn1Gd3 alloy (10^−4^–10^−7^A·cm^−2^). 

The basic electrochemical parameters obtained by Tafel extrapolation of the polarization curves are listed in Table 1. Theoretically, a higher value of corrosion potential and a lower value of corrosion current density, j_corr_, are considered to imply better corrosion resistance [20]. The results indicate that a 35 nm-thick SnO_2_ film on MgCa2Zn1 alloy shows significantly lower corrosion resistance than other coatings and is similar to that of the base alloy; corrosion potential decreases from −1.55 to −1.56 V, and the j_corr_ increases from 98 to 330 μA·cm^–2^. It does not provide effective protection against corrosion. The surface of the layer was rough and porous, as evidenced by SEM images, which could accelerate corrosion reactions. The results of the EDS analysis indicated that the tin content was lower (1.1, 1.3, and 1.6 wt.% Sn for the 35, 52.5, and 70 nm-thick films, respectively) compared to the second studied alloy (Sn content was 1.3, 2.2, and 3.0 wt.%). In addition, the substrate and coating may have undergone deterioration during the ALD process, during which the SnO₂ layer was applied at a temperature of 300 °C. For the 52.5 and 70 nm-thick films, an improvement of corrosion resistance is visible (E_corr_ increased from −1.09 to −0.4 V, and j_corr_ has decreased from the value of 4 to 3 μA·cm^−2^). The SnO_2_ thin films deposited to MgCa2Zn1Gd3 alloy improved the corrosion resistance of the uncoated alloy regardless of the thickness of the coating. Moreover, SnO_2_ 70 nm thick had higher E_corr_ and lower j_corr_ compared to SnO_2_ with the same thickness deposited on MgCa2Zn1 (E_corr_ was −0.4 and −0.22 V, and j_corr_ was 3 and 2 μA·cm^−2^ for the films deposited on MgCa2Zn1 and MgCa2Zn1Gd3 alloys, respectively). It should also be noted that the magnesium alloy with gadolinium addition had slightly better corrosion resistance compared to the alloy without Gd (Table 1). Gadolinium is distinguished by its high solubility in Mg-solid solutions at eutectic temperatures. The distribution of this element in the Mg matrix serves to reduce the corrosion rate of magnesium alloys. This phenomenon is related to the formation of a crystallographic β phase, which is resistant to corrosion [24]. 

The examples of the positive influence of SnO_2_ on corrosion resistance have been described in the literature [11,17,20,25,26]. The corrosion behavior of SnO_2_-doped dicalcium phosphate deposited on AZ31 magnesium alloy in Hank’s solution was investigated by Cui et al. [20]. The authors confirmed that corrosion current density of the coating with 5 g·dm^−3^ is 2.9 times lower than that obtained without SnO_2_. Furthermore, the coating prepared with 10 g·dm^−3^ SnO_2_ exhibited enhanced corrosion resistance relative to the alloy, likely due to its increased thickness and compactness [20]. Wang et al. [17] applied a fluorine-doped SnO_2_ coating to 317 L stainless steel in order to reduce corrosion of bipolar plates in fuel cells. They observed an improvement of corrosion resistance of SnO_2_ in a corrosive environment. Similar results on the corrosion behavior of tin dioxide films deposited on WE magnesium alloy were obtained by Jin et al. [11]. The WE alloy coated with the SnO_2_ layer reduced the corrosion current density in the SBF fluids (simulated body fluid) to 1.679 ± 0.656 μA cm^−2^ compared to the uncoated alloy, where the j_corr_ was 580.4 ± 22.4 μA∙cm^−2^, which is a 345-fold reduction. In other work [26], researchers also confirmed that the SnO_2_ coating/layer deposited on metal alloys is a good protection against corrosion in an aggressive environment, as confirmed by the lower corrosion current density and higher impedance.

**Figure 7:**
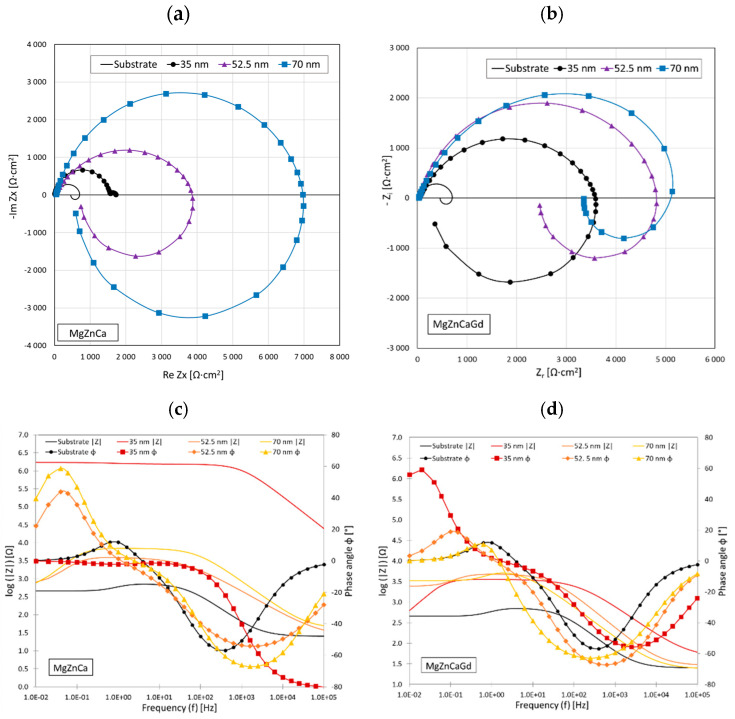
Nyquist plots for SnO_2_-coated MgCa2Zn1 (**a**) and MgCa2Zn1Gd3 (**b**) alloys and Bode plots for the same alloys (**c**,**d**) with coatings of 35 nm, 52.5 nm, and 70 nm thicknesses.

When discussing the electrochemical impedance spectroscopy (EIS) results, it is crucial to interpret the complex electrochemical behaviors observed in the SnO_2_-coated MgCa2Zn1 and MgCa2Zn1Gd3 alloys. The Nyquist plots, as shown in Figure 7a,b, provide important insights into the corrosion mechanisms of these alloys when immersed in Ringer’s solution. Both alloys show a high frequency capacitance loop on Nyquist plots related to the charge transfer reaction, often part of redox (reduction–oxidation) processes, of the surface oxide film and a low-frequency inductance loop [27]. The low-frequency inductance loop can be correlated with pitting corrosion and is related to the adsorption and desorption of intermediate species on the electrode surface and dissolution caused by chlorides [28].

The size of the loop radius, especially in the single-line Nyquist plots, is directly related to the level of corrosion resistance. Larger loop radii observed in thicker SnO_2_ coatings (i.e., 70 nm) indicate greater corrosion resistance, as they suggest a more robust and less reactive surface. Conversely, a smaller loop radii in thinner coatings (such as 35 nm) suggest a higher tendency for corrosion, indicating a less stable electrochemical interface.

**Figure 8:**
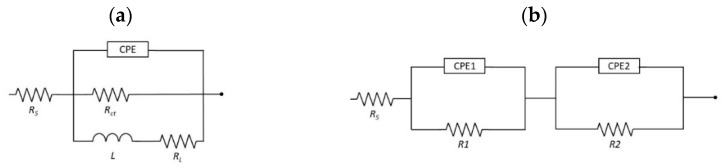
Equivalent circuits applied for analysis, where (**a**) circuit used for all samples, with exception of sample MgCa2Zn1/SnO_2_/35 nm where equivalent circuit presented on (**b**) was applied.

The electrical equivalent circuit used in the EIS analysis (Figure 8) was an inductive loop circuit used for all samples (Figure 8a), except for sample MgCa2Zn1/SnO_2_/35 nm where a circuit with two sections of constant phase element (CPE) in parallel and resistor in series was used (Figure 8b). 

The resistance of the corrosive solution is represented by R_s_, while the R_ct_ is related to the charge transfer resistance at the phase interface. An inductor (L) related to the adsorption of solution impurities and a corresponding resistance R_L_ (inductance resistance) have also been included to represent the inductive response at low frequencies. Because of the uneven distribution of the current flow due to surface irregularities, constant phase elements (CPEs) were selected over double-layer capacitance and other capacitances in the equivalent. The double-layer capacitance C_dl_ of the system was then calculated using the ‘Pseudocapacitance’ tool in EC-Lab software v. 11.41. The main corrosion resistance of the SnO_2_ coatings was assessed based on the polarization resistance of the circuits. The polarization resistance (R_p_) values, corresponding to the equivalent circuits (Figure 8), were calculated as a sum of R_ct_ and R_L_ resistances and R_1_ and R_2_, respectively, as for parallel resistors according to EEC (e.g., R_p_ = 1/(1/R_ct_ + 1/R_L_). In cases of circuits with two sections of parallel-connected capacitance and series-connected resistance (Figure 8b), the R_1_ resistance can be related to the pore resistance and refers to the resistance due to ionic conduction paths within the coating [29]. The second resistance R_2_ represents the resistance to charge transfer at the substrate/electrolyte interface R_ct_. Table 2 summarizes the parameters used to approximate the experimental EIS data for the Mg alloys with SnO_2_ coatings of different thicknesses using proposed EEC models of the pitting corrosion process. 

The double-layer capacitance (C_dl_) of a system, as derived from constant phase elements (CPEs), can be significantly influenced by the formation of corrosion products on the material’s surface. CPEs are employed to model the non-ideal capacitive behavior observed at the interface between a solid electrode and an ionic solution, which results from the separation of ionic and/or electronic charges. 

In the case of uncoated magnesium alloy surfaces, the formation of corrosion products, such as magnesium hydroxide (Mg(OH)_2_), can lead to high capacitive properties. This is because Mg(OH)_2_ tends to form a very loose, porous layer, increasing the effective surface area available for charge separation, thus enhancing the double-layer capacitance. The high C_dl_ (Table 2) observed in the case of uncoated Mg alloys indicates a less protective and more permeable layer, which might allow for continued corrosion processes. Conversely, when the surface is doped with tin oxide (SnO_2_), the capacitive properties decrease in direct proportion to the coating thickness. This is attributed to the formation of a more compact and uniform layer of corrosion product when the surface is doped with SnO₂, which reduces the effective surface area in contact with the electrolyte. The corrosion products then act as a barrier, impeding ionic movement and charge transfer, lowering the C_dl_.

The polarization resistance (R_p_) and charge transfer resistance (R_ct_) estimated in EIS tests show a proportional relationship to the thickness of the coatings tested. Increased charge transfer resistance is due to the decrease in active surface area of the support as a result of tin oxide presence. The EIS results also showed that dissolution kinetics decreased with the increased SnO_2_ coating thickness, showing an increase in diameter of the arc in Nyquist plot. The highest values were recorded for the 70 nm coating in both Mg alloys. The EIS results agree with the Tafel analysis.

**Figure 9:**
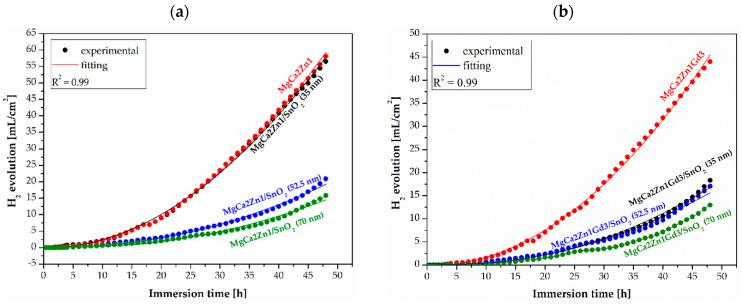
Hydrogen evolution volume as a function of immersion time in Ringer’s solution at 37 °C for 48 h for the SnO_2_ thin films applied to (**a**) MgCa2Zn1 and (**b**) MgCa2Zn1Gd3 alloys and uncoated alloys.

The corrosion activity of Mg-based alloys coated with SnO_2_ thin films was also observed during 48 h of immersion tests in Ringer’s solution at 37 °C. The corrosion resistance of the samples was monitored using a hydrogen (H_2_) evolution as an effect of corrosion products. In a study [30], it was stated that in vitro immersion tests provided in some physiological environments should give similar results to those observed in vivo. Figure 9 shows the H_2_ release during 48 h of immersion for the SnO_2_ films and uncoated alloys. 

The results of the immersion tests are similar to those of the electrochemical studies. The highest volume of H_2_ evolution was obtained for tin dioxide films deposited on MgCa2Zn1 alloy. The results obtained after 48 h of immersion were 56.53, 20.89, and 15.82 mL∙cm^−2^ for 35, 52.5, and 70 nm-thick films, respectively. The SnO_2_ deposited on Mg-based alloy with gadolinium addition was characterized by a slightly lower volume of H_2_ release. For SnO_2_ 35 nm-thick films, the hydrogen volume was 18.32 mL∙cm^−2^, and for the 52.5 and 70 nm-thick films, it was 17.04 and 12.98 mL∙cm^−2^, respectively. It should also be noted that the volume of H_2_ released for the MgCa2Zn1Gd3 alloy was lower compared to the second Mg alloy (H_2_ volume was 44.02 and 58.08 mL·cm^−2^ for MgCa2Zn1Gd3 and MgCa2Zn1, respectively). These results suggest that the magnesium alloy with Gd addition and SnO_2_ coating has an improved corrosion resistance compared with the MgCa2Zn1 coated and uncoated, which is promising for the potential use in implantology. 

**Figure 10:**
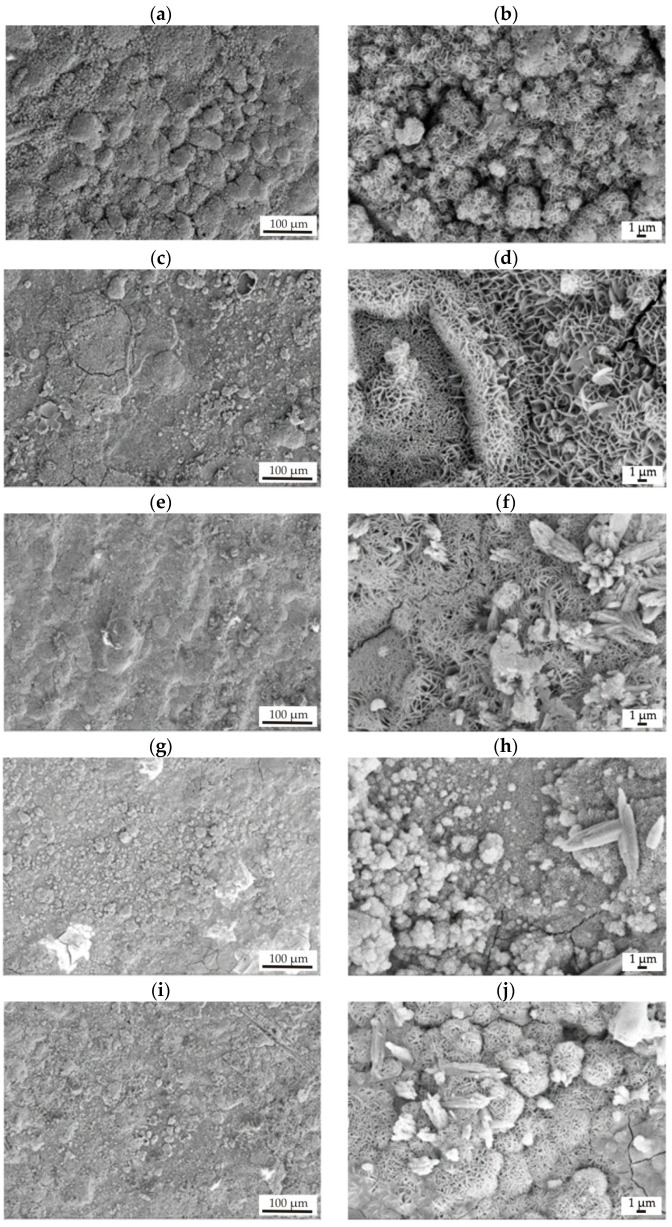

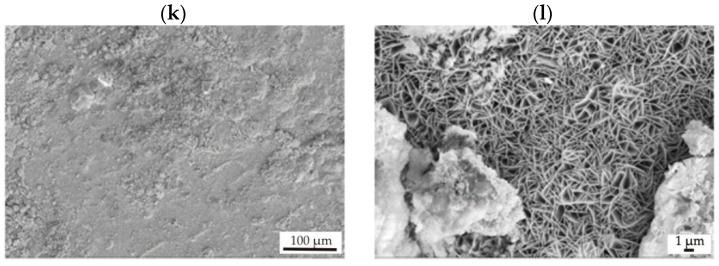
SEM images of samples’ surfaces with corrosion products of the SnO_2_ films applied onto MgCa2Zn1 alloy after (**a**,**b**) 1000 cycles, (**c**,**d**) 1500 cycles, and (**e**,**f**) 2000 cycles and MgCa2Zn1Gd3 alloy after (**g**,**h**) 1000 cycles, (**i**,**j**) 1500 cycles, and (**k**,**l**) 2000 cycles after 48 h of immersion in Ringer’s solution at 37 °C.

After 48 h of immersion in Ringer’s solution, the surfaces of the samples were examined under the microscope (Figure 10). The corrosive environment with high chloride content led to visible damage to the surface of SnO_2_ coatings, particularly in the case of MgCa2Zn1 alloy samples with 35 and 52.5 nm thick tin dioxide layers. This was confirmed by the microcracks on the surface of the samples. This is due to dehydration during the drying of the samples. It can be seen that the microcracks were smaller on the surfaces of the 70 nm-thick SnO_2_ deposited on the MgCa2Zn1 alloy. And in the case of the Mg alloy with gadolinium addition, they were not visible. It can therefore be concluded that the thickest SnO_2_ film deposited on the MgCa2Zn1Gd3 should best protect the substrate alloy against corrosion. This is consistent with the results of the immersion and electrochemical tests, where the alloy with this thin film was characterized by the lowest value of corrosion current density (j_corr_ = 2 μA·cm^−2^) and the highest value of polarization resistance (R_p_ = 54,700 Ω·cm^2^). Moreover, lamellar-shaped corrosion products were visible on the surfaces of all samples. They were denser in the case of 70 nm-thick SnO_2_ films. It was also observed that some corrosion products fell off from the surfaces of the films.

**Figure 11:**
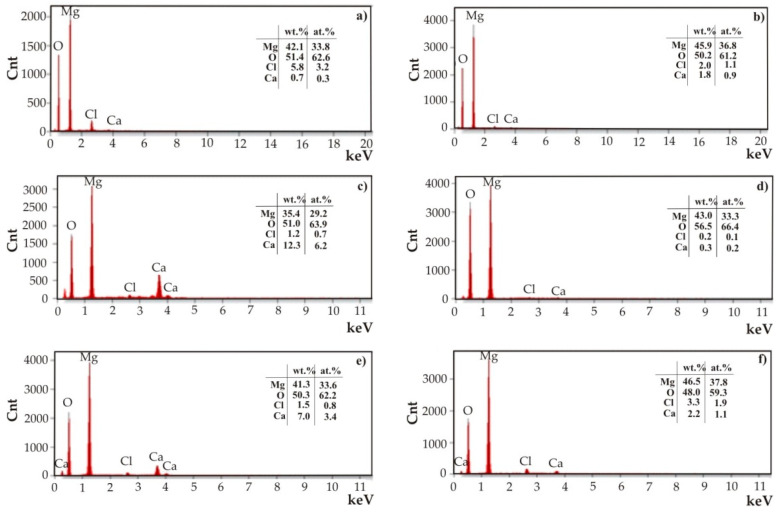
EDS analysis of corrosion products of the SnO_2_ thin films deposited onto MgCa2Zn1 alloy after (**a**) 1000 cycles, (**b**) 1500 cycles, and (**c**) 2000 cycles and MgCa2Zn1Gd3 alloy after (**d**) 1000 cycles, (**e**) 1500 cycles, and (**f**) 2000 cycles.

Observations of the corrosion products were supplemented by the EDS analysis (Figure 11). In the analyses, in addition to high intensities of reflections from oxygen and the main element of the substrate alloys (magnesium), small reflections of residues from the electrolyte in which corrosion resistance tests were performed (CaCl_2_ and NaCl) were observed. No reflections from Sn were identified, probably due to the long period of corrosion tests (48 h of immersion) for such thin nanometer layers. An evenly applied and tight coating provides initial protection against corrosion. Over time, when chloride ions from Ringer’s solution break the continuity of the nanometric protective coating, chemical reactions result in the formation of corrosion products, which also determine the corrosion resistance. In the case of this work, the SnO_2_ layers were very thin and porous. Nanopores in the films do not provide effective protection against corrosion and allow the penetration of chloride ions, leading to corrosion at the interface between the thin layer and the substrate, dissolving the protective layers. 

## 4. Conclusions

One of the methods to reduce the corrosion activity of Mg-based alloys is to deposit protective layers, including oxide layers, on their surfaces. In this work, SnO_2_ films with thicknesses of 35, 52.5, and 70 nm were deposited on MgCa2Zn1 and MgCa2Zn1Gd3 alloys using the ALD method. The effectiveness of the deposition process and the corrosion resistance of the alloys with deposited films and uncoated alloys were investigated using microstructural analysis and corrosion tests. Based on the analysis of the investigation results, the following conclusions were formulated:

1. The dominant presence of SnO_2_ rutile structure was confirmed by using XRD analysis.
2. The surface morphologies of all deposited SnO_2_ were similar. The thin films had a heterogeneous structure and a lamellar-like shape composed of nanorods. All surfaces were rough with visible pores.
3. Electrochemical and immersion tests confirmed the effectiveness of the deposited SnO_2_ thin films.
4. The values of basic corrosion parameters, the higher corrosion potentials (E_corr_), and lower corrosion current densities (j_corr_) were reported for SnO_2_-coated alloys. Moreover, SnO_2_ 70 nm-thick films deposited on MgCa2Zn1Gd3 alloy had higher E_corr_ and lower j_corr_ (E_corr_ was −0.22 V, and j_corr_ was 2 μA·cm^−2^) compared to SnO_2_ (70 nm thick) deposited on MgCa2Zn1 (E_corr_ = −0.4 V, and j_corr_ = 3 μA·cm^−2^).
5. The polarization resistance (R_p_) and charge transfer resistance (R_ct_) estimated in the EIS test show a proportional relationship to the thickness of SnO_2_ coatings on Mg alloys. Thicker coatings (i.e., 70 nm and 52.5 nm) show good protective performance in Ringer’s solution.
6. The results of the immersion tests were similar to those obtained in electrochemical studies. Hydrogen release volume measurements showed that the MgCa2Zn1Gd3 alloy coated and uncoated with SnO_2_ films had improved corrosion resistance compared to the second coated and uncoated alloy. The volume of H_2_ release for the 70 nm-thick SnO_2_ deposited on the alloy with gadolinium addition was 12.98 mL cm^−2^, and the H_2_ volume for the film with the same thickness deposited on MgCa2Zn1 was 15.82 mL∙cm^−2^.
7. Observations of the corrosion products after 48 h of immersion in Ringer’s solution showed that they had a lamellar-shape. It can also be seen that, on the surfaces of the 70 nm-thick SnO_2_ deposited on MgCa2Zn1Gd3, microcracks were not visible compared to the other samples.
8. The results of the electrochemical and immersion tests allow for the suggestion that the thickest SnO_2_ film deposited on the alloy with gadolinium addition should best protect the substrate alloy against corrosion.

## Figures and Tables

**Table 1:** Corrosion parameters of SnO_2_ thin films and the MgCa2Zn1 and MgCa2Zn1Gd3 alloys.

Sample	Corrosion Potential, E_corr_, V	Polarization Resistance, R_p_, Ω·cm^2^	Corrosion Current Density, j_corr_, μA·cm^–2^
MgCa2Zn1	−1.55	200	98
MgCa2Zn1/SnO_2_/35 nm	−1.56	30	330
MgCa2Zn1/SnO_2_/52.5 nm	−1.09	41,000	4
MgCa2Zn1/SnO_2_/70 nm	−0.4	49,000	3
MgCa2Zn1Gd3	−1.56	363.1	57
MgCa2Zn1Gd3/SnO_2_/35 nm	−0.5	21,000	6
MgCa2Zn1Gd3/SnO_2_/52.5 nm	−0.32	39,400	4
MgCa2Zn1Gd3/SnO_2_/70 nm	−0.22	54,700	2

**Table 2:** Parameter values obtained from the EIS data fitting for the MgCa2Zn1 and MgCa2Zn1Gd3 alloys coated with SnO_2_ thin films.

Sample	Rs	CPE	a	C_dl_	R_ct_/R_1_	CPE	a	C_dl_	L	R_L_/R_2_	R_p_
	Ω·cm^2^	µF·s^(a−1)^ cm^−2^	-	µF·cm^−2^	kΩ·cm^2^	µF·s^(a−1)^ cm^−2^	-	µF cm^−2^	kH cm^2^	kΩ·cm^2^	kΩ·cm^2^
MgCa2Zn1 alloy											
Substrate	25.2	10.91	0.85	4.67	0.71	-	-	-	0.25	1.10	0.43
SnO_2_/35 nm	13.3	0.24	0.90	0.10	1.56	1.82 × 10^–3^	0.79	1.33 × 10^–3^	-	0.17	0.15
SnO_2_/52.5 nm	23.6	5.32	0.69	9.07	4.00	-	-	-	9.63	0.84	0.70
SnO_2_/70 nm	41.8	7.45	0.84	4.05	7.02	-	-	-	9.09	0.55	0.51
MgCa2Zn1Gd3 alloy											
Substrate	21.0	13.19	0.89	7.77	0.75	-	-	-	0.25	0.98	0.42
SnO_2_/35 nm	44.7	1.81	0.74	3.16	3.59	-	-	-	9.63	0.24	0.23
SnO_2_/52.5 nm	28.8	2.73	0.84	1.20	4.90	-	-	-	9.20	4.80	2.42
SnO_2_/70 nm	23.5	8.85	0.77	3.75	6.20	-	-	-	2.00	7.20	3.33

## Data Availability

The original contributions presented in the study are included in the article, further inquiries can be directed to the corresponding author.
